# Detection of curcumin and its metabolites in hepatic tissue and portal blood of patients following oral administration

**DOI:** 10.1038/sj.bjc.6601623

**Published:** 2004-03-02

**Authors:** G Garcea, D J L Jones, R Singh, A R Dennison, P B Farmer, R A Sharma, W P Steward, A J Gescher, D P Berry

**Affiliations:** 1 Cancer Biomarkers and Prevention Group, Departments of Cancer Studies and Biochemistry, University of Leicester, 5th Floor Robert Kilpatrick Clinical Sciences Building, Leicester LE2 7LX, UK; 2 Department of Hepatobiliary Surgery, The Leicester General Hospital, University Hospitals of Leicester, Gwendolen Road, Leicester LE5 4PW, UK

**Keywords:** curcumin, chemoprevention, human, pharmacokinetics

## Abstract

Studies *in vitro* and in animal models of colorectal and hepatocellular cancers suggest that curcumin is an effective chemopreventive agent. In this pilot trial, we investigated whether oral administration of curcumin results in concentrations of the agent in normal and malignant human liver tissue, which are sufficient to elicit pharmacological activity. In total, 12 patients with hepatic metastases from colorectal cancer received 450–3600 mg of curcumin daily, for 1 week prior to surgery. Levels of curcumin and its metabolites were measured by HPLC in portal and peripheral blood, bile and liver tissue. Curcumin was poorly available, following oral administration, with low nanomolar levels of the parent compound and its glucuronide and sulphate conjugates found in the peripheral or portal circulation. While curcumin was not found in liver tissue, trace levels of products of its metabolic reduction were detected. In patients who had received curcumin, levels of malondialdehyde-DNA (M_1_G) adduct, which reflect oxidative DNA changes, were not decreased in post-treatment normal and malignant liver tissue when compared to pretreatment samples. The results suggest that doses of curcumin required to furnish hepatic levels sufficient to exert pharmacological activity are probably not feasible in humans.

Curcumin [1,7-bis(4-hydroxy-3-methoxyphenyl)-1,6-heptadien-3,5-dione] is a potent antioxidant derived from the spice turmeric. It exerts cancer chemopreventive efficacy in a wide variety of rodent models of carcinogenesis (Kelloff *et al*, 1994). In common with several other diet-derived polyphenols, curcumin has low systemic bioavailability (Ireson *et al*, 2001). This pharmacokinetic feature of curcumin, which has been observed across several species, is the result of poor absorption and avid metabolic conjugation and reduction (Ireson *et al*, 2002; Sharma *et al*, submitted for publication). Despite the evidence that curcumin is poorly available following oral administration, there are reports that curcumin at 50–200 mg kg^−1^ doses exerts biological activity on sites distant from the locus of absorption in rodents, such as breast (Inano and Onoda, 2002), prostate (Frank *et al*, 2003), lung (Menon *et al*, 1995) and especially the liver (Busquets *et al*, 2001; Park *et al*, 2001; Nanji *et al*, 2003). Studies on gall bladder contractility following oral curcumin in humans suggest that curcumin may exert biological actions at doses of 20 mg (Rasyid & Lelo 1999; Rasyid *et al*, 2002).

Curcumin is thought to exert its chemopreventive efficacy via mechanisms including antioxidation and prevention of oxidative DNA damage. In *Apc*^Min+^ mice, a model of familial adenomatous polyposis, dietary curcumin reduced adenoma burden (Perkins *et al*, 2002b), which was accompanied by a reduction in adenoma levels of the oxidative DNA adduct (2-deoxy-*β*-dierythropentafuranosyl)pyrimido[1,2-*a*]-purin-10(3H)-one (malondialdehyde-DNA adduct, M_1_G) (Perkins *et al*, 2002a). Other mechanisms by which curcumin may exert chemopreventive action are interference with the transcription of the enzyme cyclooxygenase-2 (COX-2) (Plummer *et al*, 1999), induction of apoptosis (Kuo *et al*, 1996; Kawamori *et al*, 1999) and antiangiogenesis (Arbiser *et al*, 1998). The concentrations of curcumin needed to exert these effects in cells *in vitro*, range from 5 to 50 *μ*M (Sharma, 1976; Kunchandy and Rao, 1990; Huang *et al*, 1991; Tonessan and Greenhill, 1992; Reddy and Lokesh, 1992; Subramanian *et al*, 1994; Plummer *et al*, 1999). Little is known about the potential pharmacological efficacy of products of the metabolic conjugation and reduction of curcumin. Curcumin metabolites were much less capable of suppressing COX-2 transcription in cells *in vitro* than the parent compound (Ireson *et al*, 2002), indicating that chemopreventive efficacy may be in the main mediated by curcumin *per se* and that metabolic conjugation and reduction constitute pharmacological deactivation steps.

The disposition of curcumin in liver tissue, portal circulation and bile is unknown in humans. Therefore, we aimed to determine the levels of curcumin achievable in human liver following oral administration of curcumin. In particular, the hypothesis was tested that oral consumption of curcumin could produce liver tissue concentrations in humans consistent with levels shown to exert pharmacological action *in vitro*. These data could help to assess the feasibility of using curcumin as part of a strategy to prevent the development of hepatic metastases following resection of primary colorectal adenocarcinomas.

## METHODS

### Patients

A total of 12 patients (four female, eight male, aged 44–76 years) with hepatic metastatic disease from primary colorectal adenocarcinomas, were recruited into the trial following approval by the local ethics committee. All patients gave written informed consent for their tissue to be used in the trial. Their haematological profiles, urea and electrolytes and hepatic function were all within normal range, as defined by the clinical laboratories of the University Hospitals of Leicester. Four of the patients had received preoperative chemotherapy, consisting of a 3-month course of 5-fluorouracil and folinic acid. Their drug history included antihypertensives (five patients), diuretics (two patients) and simple analgesics (five). Pretreatment tissue samples of normal and malignant liver tissue were obtained at staging laparoscopy, which was performed to exclude peritoneal disease undetectable by computed tomography, prior to the patient's resection surgery. The curcumin content in the capsules was ascertained by HPLC. Patients (four per dose level) received a purified turmeric extract formulated in capsules obtained from Sabinsa Corp (‘Curcumin C3 complex’, Piscataway, NJ, USA). Each capsule contained 450 mg curcumin, 30 mg of desmethoxycurcumin and 20 mg of bidesmethoxycurcumin. Dose levels were 1, 4 or 8 capsules daily, translating to 450, 1800 or 3600 mg of curcumin, for 1 week prior to surgery. Samples of peripheral blood were taken 1 h immediately after curcumin dosing, and hepatic resection was performed 6–7 h after the last dose of curcumin. Samples of portal blood, bile and further samples of peripheral blood were taken intraoperatively. Blood and tissue samples were stored for up to 10 weeks at −80°C prior to analysis.

### HPLC analysis and mass spectrometry

Authentic curcumin glucuronide and curcumin sulphate were generated by incubation with rat liver microsomes and cytosol respectively, with appropriate cofactors (Ireson *et al*, 2001). Authentic hexahydrocurcumin and hexahydrocurcuminol, products of the metabolic reduction of curcumin, were generated as a mixture by reacting curcumin with sodium borohydride (Ireson *et al*, 2001). The identity of the products of chemical reduction of curcumin was validated by tandem mass spectrometry, which confirmed the presence of the respective molecule ions (hexahydrocurcumin *m*/*z*=373, hexahydrocurcuminol *m*/*z*=375). These species were not separated further and were available for comparison only in impure form.

Tissues were thawed, homogenised in ammonium acetate buffer (1 M, pH 4.6, 10% w v^−1^) and extracted with ethylacetate, which had been presaturated with ice-cold propan-2-ol. The extract was analysed for the presence of curcumin, its two metabolic conjugates curcumin sulphate and curcumin glucuronide and its metabolic reduction products hexahydrocurcumin and hexahydrocurcuminol by HPLC (UV detection) as described by Ireson *et al* (2001). The validation of the analytical method for curcumin and the curcumin conjugates has been described by Ireson *et al* (2001). Curcumin and its conjugates were detected at 420 nm, the products of curcumin reduction at 280 nm. Under the extraction and assay conditions, the limit of detection for curcumin and its conjugates was approximately 3 nM. In separate experiments, the recovery of curcumin was determined to be 80.7±2.3% from plasma, 55.3±10.2% from bile and 60.3±4.3% and 65.8±5.3% from malignant and normal liver tissue, respectively (mean±s.d., *n*=4). We have established earlier that the recovery of curcumin glucuronide and curcumin sulphate from rat liver tissue is poor, between 40 and 50% (Ireson, 2002). Absence of pure hexahydrocurcumin and hexahydrocurcuminol confounded reliable determination of their recovery. Identification was performed by co-chromatography using authentic metabolic standards and, whenever possible, by mass spectrometry using a Quattro Ultima Platinum Mass Spectrometer (Waters, Manchester, UK). For mass spectral analysis, samples (10 *μ*l) of eluate obtained from the HPLC column containing suspected curcumin-derived species were dried and solubilized in acetonitrile : water (7 : 3) and injected into the mass spectrometer using a back flow of 70/30 acetonitrile/water at 50 *μ*l min^−1^ (Waters Alliance 2695 HPLC pump) using desolvation and source temperatures of 200 and 120°C, respectively. The mass spectrometer was tuned up to each authentic agent prior to sample analysis. Analysis involved a scan of 40–600*m*/*z*. Tandem mass spectrometry was used to fragment molecular ions into product ions, argon was the collision gas. The determination of molecular species related to curcumin proved consistently difficult due to inherent poor sensitivity, which may be, at least in part, a function of the negative ion polarity of the analyte species. Therefore, it was not feasible to confirm hexahydrocurcumin and hexahydrocurcuminol peak identity in biological matrix extracts by mass spectrometry. On gross extrapolation, UV spectrophotometry was at least 10-fold more sensitive than mass spectrometry as method of HPLC detection for these species.

### Stability of curcumin and its metabolites

In the light of the fact that levels of detected analyte species were consistently low, we ascertained that curcumin, its glucuronide and sulphate conjugates and reduction products hexahydrocurcumin and hexahydrocurcuminol were sufficiently stable under storage and assay conditions to allow meaningful interpretation of analysis results. The stability was checked under conditions of tissue storage at −80°C (spiked tissue samples) and of the HPLC assay at room temperature. After 3 months of storage, areas of peaks indicative of curcumin and its metabolites in extracts from liver tissue were unchanged. When kept after dissolution in HPLC injection solvent (acetonitrile : water 1 : 1) curcumin and its metabolites were stable for at least 12 h at room temperature.

### Analysis of M_1_G adduct levels

Extraction of genomic DNA from liver tissue obtained at staging laparoscopy and during surgery, and analysis of M_1_G adduct levels by immunoslotblot was performed as described previously by Leuratti *et al* (1998) using a primary anti-M_1_G antibody provided by Dr Lawrence Marnett (Vanderbilt University, TN, USA). Discrepancies in the amount of DNA in each slot were corrected for by staining the nitrocellulose filter with propidium iodide and performing UV light densitometry. The limit of detection for M_1_G was five adducts per 10^8^ nucleotides.

## RESULTS

**Figure 1:**
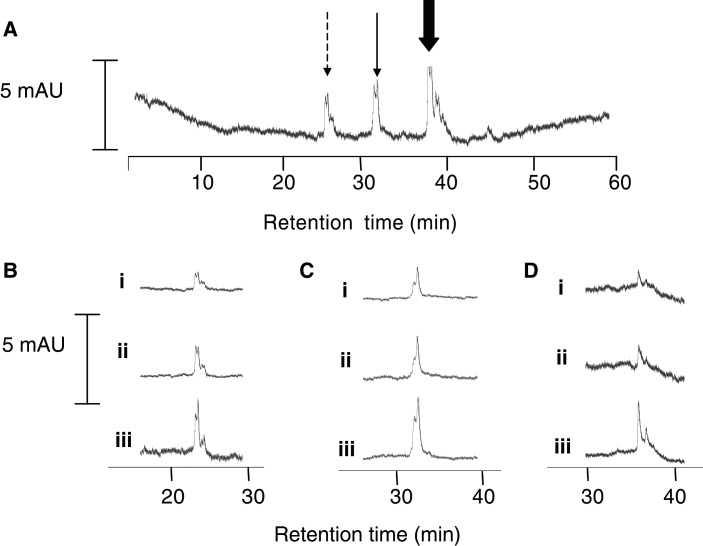
HPLC analyses of an extract of (**A**) portal blood of a patient who received curcumin 3600 mg daily for a week; (**B**, i) an extract of HPLC solvent eluting with the peak with retention time 25 min in (**A**) (broken arrow), (**B**, ii) a solution of authentic curcumin glucuronide and (**B**, iii) a mixture of i and ii; (**C**, i) an extract of HPLC solvent eluting with the peak with retention time 31 min in (**A**) (solid arrow), (**C**, ii) a solution of authentic curcumin sulphate, and (**C**, iii) a mixture of i and ii; (**D**, i) an extract of HPLC solvent eluting with the peak with retention time 37 min in (**A**) (block arrow), (**D**, ii) a solution of authentic curcumin, and (**D**, iii) a mixture of i and ii. Detection was by UV spectrophotometry at 420 nm. It is important to point out that the peaks of curcumin and curcumin conjugates are accompanied by peaks of the respective desmethoxy analogues at lower abundance, which is a corollary of the fact that desmethoxycurcumin and bisdesmethoxycurcumin are inevitable constituents of the curcumin formulation (at 10%, Ireson *et al*, 2001). The indication of presence of peaks for desmethoxycurcumin, desmethoxycurcumin glucuronide and desmethoxycurcumin sulphate accompanying the respective peaks of the curcumin-derived species is consistent with the inferences as to peak identity. ‘mAU’ denotes milli absorbance units. Surgery was conducted 6 h after the last of seven daily doses of curcumin. The chromatograms that are from one patient are representative of three of the four patients who received curcumin at 3600 mg. For details of surgery, sample preparation and HPLC analysis, see Materials and methods.

HPLC analysis of the portal serum of three patients who had received 3600 mg curcumin furnished three peaks. These peaks were tentatively characterised as curcumin, curcumin glucuronide and curcumin sulphate on the basis of their retention times. The peak areas were below the limit of quantitation and near the limit of detection (∼3 nM). Unambiguous identification of these three species was achieved by collection of the eluent at the retention times of the respective peaks, evaporation of solvent and reanalysis of the residues by co-chromatography using authentic curcumin, curcumin glucuronide and curcumin sulphate (Figure 1

). Mass spectrometric analysis corroborated the chromatographic evidence of peak identity, yielding molecule ions of *m/z* 543, 447 and 367 for curcumin glucuronide, curcumin sulphate and curcumin, respectively. A peak coeluting with curcumin was also found in the peripheral circulation in two patients who had received the last dose of curcumin 3600 mg 1 h prior to blood collection. Analysis of peripheral and portal blood from patients who had received 1800 or 450 mg of curcumin did not reveal evidence for the presence of curcumin or its conjugates.

**Figure 2:**
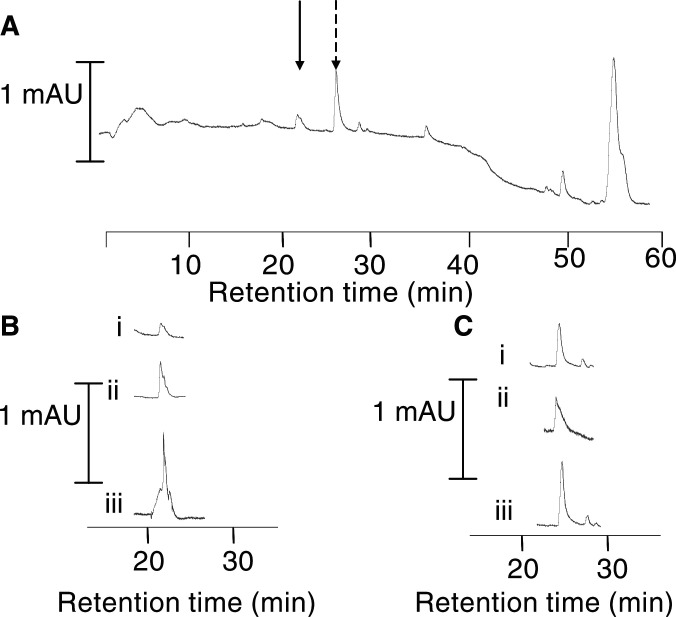
HPLC analyses of extract of (**A**) normal liver tissue from a patient who had received 3600 mg of curcumin; (**B**, i) an eluate of the peak suspected to be hexahydrocurcuminol (solid arrow), (**B**, ii) chemically generated hexahydrocurcuminol at a concentration close to its detection limit, and (**B**, iii) a mixture of i and ii; (**C**, i) an eluate of the peak suspected to be hexahydrocurcumin (broken arrow), (**C**, ii) chemically generated hexahydrocurcumin at a concentration close to its detection limit, and (**C**, iii) a mixture of i and ii. Detection was by UV spectrophotometry at 280 nm. ‘mAU’ denotes milliabsorbance units. For details of surgery, sample preparation, HPLC analysis and chemical reduction of curcumin to hexahydrocurcuminol and hexahydrocurcumin, see Materials and methods.

Curcumin, curcumin sulphate and curcumin glucuronide were not found in bile or normal and malignant liver tissue in any of the patients who had received curcumin. Analysis of normal liver tissue from one patient who had received 3600 mg of curcumin daily yielded two peaks with retention times of 22 and 27 min, which indicates the presence of hexahydrocurcumin and hexahydrocurcuminol, products of the metabolic reduction of curcumin. The identity of these species was further defined after isolation of material eluting with the peaks and its reanalysis by co-chromatography with a mixture of hexahydrocurcumin and hexahydrocurcuminol generated by chemical reduction of curcumin (Figure 2

). The relatively poor sensitivity of mass spectrometry as a means of detection of hexahydrocurcumin and hexahydrocurcuminol confounded mass spectral verification of peak identity.

Next, we investigated whether dietary curcumin affects levels of oxidative DNA adducts as reflected by M_1_G in normal and malignant liver tissue. Levels measured in four patients who received curcumin 3600 mg were as follows (expressed as adducts per 10^7^ nucleotides): 4.3±0.4 in normal tissue obtained during staging laparoscopy (pretreatment), 6.3±3.8 in normal surgical tissue (post-treatment), 2.5±1.5 in pretreatment malignant tissue and 6.3±5.2 in post-treatment malignant surgical tissue. The difference between pre- and post-treatment values was significant (*P*<0.05 by Student's *t*-test). A very similar difference in M_1_G levels between pre- and post-treatment samples was observed in patients who received 450 or 1800 mg curcumin. For orientation, these values compare with a mean of 4.5 adducts per 10^7^ nucleotides reported in normal rat liver (Sharma *et al*, 2001a) for orientation, these values compose with a mean of 4.5 adducts per 10^7^ nucleotides reported in rat liver (Sharma *et al*, 2001a) and with 5–10 adducts per 10^7^ nucleotides in healthy liver tissue from humans (Chaudhary *et al*, 1994). The fact that levels were consistently increased post-treatment irrespective of curcumin dose is counterintuitive in the light of the absence of curcumin-derived species in the livers of patients on the low dose, and its presence at extremely low levels in only one of the patients on 3600 g curcumin (see above). Therefore, one might expect *a priori* either that there is no difference in M_1_G levels between pre- and post-treatment samples, or that, if curcumin was present at sufficient amounts, M_1_G levels are decreased post-treatment. In the light of all these considerations, we surmise that the observed difference in oxidative DNA adduct levels between pre- and post-treatment samples is unrelated to curcumin. It is conceivable that the difference was caused using the Pringle manoeuvre during post-treatment surgery. This procedure, which is widely used during liver resection to reduce bleeding, involves occlusion of the hepatic artery and portal vein. Consequently, Kupffer cells are activated, resulting in the production of reactive oxygen species and cytokines, thus precipitating oxidative stress (Colletti *et al*, 1990; Wang *et al*, 1994). While we did not further investigate a possible link between Pringle manoeuvre and rise in M_1_G levels in patients who did not receive curcumin, our suspicion of an association was supported by a gross correlation between these levels and the duration of application of the Pringle clamp.

## DISCUSSION

The results presented above provide the first evidence in humans that oral administration of curcumin furnishes trace levels of the parent compound and its metabolites in the liver and portal circulation. However, the levels of curcumin recovered were below 10^−8^ M, thus of an order of magnitude that is unlikely to exert pharmacological effects in the light of results obtained in experiments in which cells *in vitro* were exposed to curcumin (see e.g., Sharma, 1976; Kunchandy and Rao, 1990; Huang *et al*, 1991; Reddy and Lokesh, 1992; Tonessan and Greenhill, 1992; Subramanian *et al*, 1994; Plummer *et al*, 1999).

The lack of quantifiable levels of curcumin in plasma is consistent with recent clinical reports in which doses of up to 180 mg of curcumin failed to establish detectable plasma levels (Sharma *et al*, 2001b) and very high doses (up to 8 g) yielded curcumin peak levels of only approximately 0.5–2 *μ*M within 1 h of oral administration (Cheng *et al*, 2001). Traces of conjugated curcumin metabolites were detected in the urine of humans who had received doses of 1800–3600 mg daily (Sharma *et al*, submitted for publication). The presence of curcumin conjugates in the portal circulation is consistent with reports that curcumin undergoes glucuronidation and sulphation in the intestine of both humans and rodents (Ireson *et al*, 2001). We have recently shown that oral consumption of up to 3600 mg curcumin leads to curcumin concentrations of 10 nmol g^−1^ tissue in human colorectal mucosa, levels that might be high enough to exert biological efficacy (Garcea *et al*, submitted). However, only trace levels of the parent compound were found in the peripheral circulation.

Considerable interest exists in the use of curcumin as a chemopreventive agent (Kelloff *et al*, 1994). The results of the analytical determination presented here render it unlikely that curcumin holds any potential in the chemoprevention of hepatic malignancies. Consistent with this notion, curcumin did not reduce levels of M_1_G adducts in normal or malignant hepatic tissue at any of the doses used in this study. Nevertheless, this latter conclusion has to be drawn with utmost caution in the light of the unexpected increase in M_1_G adduct levels post-treatment, which was probably unrelated to treatment with curcumin and might have been rather a function of the surgical procedure used in the generation of post-treatment tissue. For comparison, dietary curcumin (2%) reduced M_1_G levels in colon mucosa of rats that had been challenged with carbon tetrachloride, an oxygen radical-generating hepatotoxicant, but failed to affect M_1_G levels in rat liver tissue (Sharma *et al*, 2001a). Furthermore, at 0.2% in the diet curcumin decreased M_1_G levels in intestinal adenomas of *Apc*^Min+^ mice (Perkins *et al*, 2002a).

The poor availability of curcumin in humans shown here impacts also on the feasibility of its role as a modulator of chemotherapy-induced apoptosis in tissues remote from the gastrointestinal tract, as discussed recently (Somasundaram *et al*, 2002; Mitchell, 2003). In conclusion, clinical trials of curcumin should focus on the prevention of colorectal tumours but not tumours distant from the locus of absorption such as the liver.
